# Supplementary material for: Virulence characteristics of Blumeria graminis f. sp. tritici and its genetic diversity by EST-SSR analyses
Source: PeerJ. 2022 Oct 14;10:e14118. doi: 10.7717/peerj.14118 (PMC9575677; doi:10.7717/peerj.14118)
Supplement: Supplemental Information 1 [file peerj-10-14118-s001.doc]

Supplemental Table 1. Collection sites, date, location, and cultivars crop growth stages for all *Blumeria graminis* f. sp. *tritici* isolates used in this study

| No. | Isolate | Collection date | Collection site | Collection location | Cultivars |
| --- | --- | --- | --- | --- | --- |
| 1 | L1 | 21-Jun-15 | Anshan, Liaoning | 122.°86’E, 41°21’N | Liaochun 18 |
| 2 | L2 | 21-Jun-15 | Anshan, Liaoning | 122.°86’E, 41°21’N | Liaochun 18 |
| 3 | L14 | 22-Jun-15 | Fuxin, Liaoning | 121°74’E, 41°95’N | Advanced Lines |
| 4 | L17 | 22-Jun-15 | Fuxin, Liaoning | 121°74’E, 41°95’N | Liaochun 15 |
| 5 | L18 | 22-Jun-15 | Fuxin, Liaoning | 121°74’E, 41°95’N | Liaochun 15 |
| 6 | L19 | 22-Jun-15 | Fuxin, Liaoning | 121°74’E, 41°95’N | Liaochun 15 |
| 7 | L21 | 18-Jun-15 | Shenyang, Liaoning | 123°19’E, 41°65’N | Liaochun 18 |
| 8 | L23 | 18-Jun-15 | Shenyang, Liaoning | 123°19’E, 41°65’N | Liaochun 18 |
| 9 | L24 | 18-Jun-15 | Shenyang, Liaoning | 123°57’E, 41°84’N | Trapping nursery |
| 10 | L25 | 18-Jun-15 | Shenyang, Liaoning | 123°57’E, 41°84’N | Shenmian 2135 |
| 11 | L27 | 18-Jun-15 | Shenyang, Liaoning | 123°57’E, 41°84’N | Shenmian 2137 |
| 12 | L29 | 18-Jun-15 | Shenyang, Liaoning | 123°57’E, 41°84’N | Chinese Spring |
| 13 | L30 | 18-Jun-15 | Shenyang, Liaoning | 123°57’E, 41°84’N | Line A |
| 14 | L31 | 18-Jun-15 | Shenyang, Liaoning | 123°57’E, 41°84’N | Reliance |
| 15 | L33 | 18-Jun-15 | Shenyang, Liaoning | 123°57’E, 41°84’N | Mianyang 28 |
| 16 | L34 | 18-Jun-15 | Shenyang, Liaoning | 123°57’E, 41°84’N | Avocet S |
| 17 | L35 | 20-Jun-15 | Kangping, Liaoning | 123°57’E, 41°84’N | Liaochun 18 |
| 18 | L38 | 20-Jun-15 | Kangping, Liaoning | 123°26’E, 42°88’N | Liaochun 18 |
| 19 | L39 | 20-Jun-15 | Kangping, Liaoning | 123°26’E, 42°88’N | Liaochun 18 |
| 20 | L42 | 20-Jun-15 | Kangping, Liaoning | 123°26’E, 42°88’N | Liaochun 25 |
| 21 | L44 | 20-Jun-15 | Kangping, Liaoning | 123°26’E, 42°88’N | Liaochun 25 |
| 22 | L45 | 20-Jun-15 | Kangping, Liaoning | 123°26’E, 42°88’N | Local variety, breeding parent nursery |
| 23 | L48 | 20-Jun-15 | Kangping, Liaoning | 123°26’E, 42°88’N | Local variety, breeding parent nursery |
| 24 | L50 | 24-Jun-15 | Xinmin, Liaoning | 122°75’E, 45°85’N | Liaochun 18 |
| 25 | L59 | 24-Jun-15 | Xinmin, Liaoning | 122°75’E, 45°85’N | Liaochun 18 |
| 26 | L60 | 24-Jun-15 | Xinmin, Liaoning | 122°75’E, 45°85’N | Liaochun 18 |
| 27 | L61 | 24-Jun-15 | Xinmin, Liaoning | 122°75’E, 45°85’N | Trapping nursery |
| 28 | L62 | 24-Jun-15 | Xinmin, Liaoning | 122°75’E, 45°85’N | Trapping nursery |
| 29 | L64 | 24-Jun-15 | Xinmin, Liaoning | 122°75’E, 45°85’N | Trapping nursery |
| 30 | H1 | 4-Jul-15 | Harbin, Heilongjiang | 126°47’E, 48°02’N | Volunteer wheat |
| 31 | H2 | 4-Jul-15 | Harbin, Heilongjiang | 126°47’E, 48°02’N | Kechun 9 |
| 32 | H3 | 4-Jul-15 | Harbin, Heilongjiang | 126°47’E, 48°02’N | Trapping nursery |
| 33 | H4 | 4-Jul-15 | Harbin, Heilongjiang | 126°47’E, 48°02’N | Trapping nursery |
| 34 | H5 | 4-Jul-15 | Harbin, Heilongjiang | 126°47’E, 48°02’N | Advanced Lines |
| 35 | H7 | 4-Jul-15 | Harbin, Heilongjiang | 126°47’E, 48°02’N | Longmai 34 |
| 36 | H9 | 6-Jul-15 | Keshan, Heilongjiang | 125°84’E, 48°02’N | Advanced Lines |
| 37 | H10 | 6-Jul-15 | Keshan, Heilongjiang | 125°84’E, 48°02’N | Kechun 110217 |
| 38 | H12 | 6-Jul-15 | Keshan, Heilongjiang | 125°84’E, 48°02’N | Chancellor |
| 39 | H16 | 6-Jul-15 | Keshan, Heilongjiang | 125°84’E, 48°02’N | Kechun 11362 |
| 40 | H17 | 6-Jul-15 | Keshan, Heilongjiang | 125°84’E, 48°02’N | Kehua (Parental nursery) |
| 41 | H18 | 6-Jul-15 | Jiushan, Heilongjiang | 125°58’E, 48°99’N | Volunteer wheat |
| 42 | H19 | 6-Jul-15 | Jiushan, Heilongjiang | 125°58’E, 48°99’N | Volunteer wheat |
| 43 | H21 | 6-Jul-15 | Jiushan, Heilongjiang | 125°58’E, 48°99’N | Longken 3 |
| 44 | H22 | 6-Jul-15 | Jiushan, Heilongjiang | 125°58’E, 48°99’N | Afu |
| 45 | H28 | 6-Jul-15 | Jiushan, Heilongjiang | 125°58’E, 48°99’N | Kehua (Parental nursery) |
| 46 | H29 | 7-Jul-15 | Beian, Heilongjiang | 126°54’E, 48°32’N | Advanced Lines |
| 47 | H30 | 7-Jul-15 | Beian, Heilongjiang | 126°54’E, 48°32’N | Longmai 26 |
| 48 | H31 | 7-Jul-15 | Beian, Heilongjiang | 126°54’E, 48°32’N | Longmai 26 |
| 49 | H36 | 7-Jul-15 | Beian, Heilongjiang | 126°54’E, 48°32’N | Local variety, breeding parent nursery |
| 50 | H37 | 5-Jul-15 | Hongxinglong, Heilongjiang | 131°58’E, 46°70’N | Advanced Lines |
| 51 | H38 | 5-Jul-15 | Hongxinglong, Heilongjiang | 131°58’E, 46°70’N | Hong 2011-709 |
| 52 | H40 | 5-Jul-15 | Hongxinglong, Heilongjiang | 131°58’E, 46°70’N | Little Club nursery |
| 53 | H44 | 5-Jul-15 | Hongxinglong, Heilongjiang | 131°58’E, 46°70’N | Nongda 11-1157 |
| 54 | H45 | 5-Jul-15 | Hongxinglong, Heilongjiang | 131°58’E, 46°70’N | Advanced Lines |
| 55 | H46 | 5-Jul-15 | Hongxinglong, Heilongjiang | 131°58’E, 46°70’N | Nongda 11-1157 |
| 56 | C1-2 | 2-May-15 | Guangyuan, Sichuan | 105°86’E, 32°41’N | Mianmai 39 |
| 57 | C1-3 | 2-May-15 | Guangyuan, Sichuan | 105°86’E, 32°41’N | Mianmai 40 |
| 58 | C2-1 | 2-May-15 | Guangyuan, Sichuan | 105°86’E, 32°41’N | Chuanmai 45 |
| 59 | C2-3 | 2-May-15 | Guangyuan, Sichuan | 105°86’E, 32°41’N | Chuanmai 45 |
| 60 | C2-4 | 2-May-15 | Guangyuan, Sichuan | 105°86’E, 32°41’N | Chuanmai 46 |
| 61 | C3-1 | 3-May-15 | Mianyang, Sichuan | 104°88’E, 31°91’N | Breeding parent nursery |
| 62 | C5-1 | 3-May-15 | Mianyang, Sichuan | 104°88’E, 31°91’N | Mianyang 28 |
| 63 | C6-1 | 3-May-15 | Mianyang, Sichuan | 104°88’E, 31°91’N | Mianyang 28 |
| 64 | C6-2 | 3-May-15 | Mianyang, Sichuan | 104°88’E, 31°91’N | Mianmai 51 |
| 65 | C10-2 | 3-May-15 | Mianyang, Sichuan | 104°88’E, 31°91’N | Mianyang 28 |
| 66 | C11 | 4-May-15 | Guanghan, Sichuan | 104°27’E, 30°97’N | Armada |
| 67 | C11-1 | 4-May-15 | Guanghan, Sichuan | 104°27’E, 30°97’N | Mianyang 28 |
| 68 | C13-3 | 4-May-15 | Guanghan, Sichuan | 104°27’E, 30°97’N | Line E |
| 69 | C13-4 | 4-May-15 | Guanghan, Sichuan | 104°27’E, 30°97’N | Chiyacu, Trapping nursery |
| 70 | C13-5 | 4-May-15 | Guanghan, Sichuan | 104°27’E, 30°97’N | Chiyacu, Trapping nursery |
| 71 | C14 | 4-May-15 | Guanghan, Sichuan | 104°27’E, 30°97’N | Little Club nursery |
| 72 | C17 | 4-May-15 | Guanghan, Sichuan | 104°27’E, 30°97’N | Little Club nursery |
| 73 | C18 | 5-May-15 | Chengdu, Sichuan | 104°17’E, 30°48’N | Chuan 3023 |
| 74 | C19 | 5-May-15 | Chengdu, Sichuan | 104°17’E, 30°48’N | Chuanyu 24 |
| 75 | C31 | 5-May-15 | Chengdu, Sichuan | 104°17’E, 30°48’N | Mianmai 51 |
| 76 | C34 | 5-May-15 | Chengdu, Sichuan | 104°17’E, 30°48’N | Mianmai 51 |
| 77 | C35 | 5-May-15 | Guangan, Sichuan | 106°54’E, 30°45’N | Local variety |
| 78 | C36 | 5-May-15 | Guangan, Sichuan | 106°54’E, 30°45’N | Chuannong 25 |
| 79 | C37 | 5-May-15 | Guangan, Sichuan | 106°54’E, 30°45’N | Chuanyu 24 |
| 80 | C39 | 5-May-15 | Guangan, Sichuan | 106°54’E, 30°45’N | Local variety |
