# Supplementary material for: Virulence characteristics of Blumeria graminis f. sp. tritici and its genetic diversity by EST-SSR analyses
Source: PeerJ. 2022 Oct 14;10:e14118. doi: 10.7717/peerj.14118 (PMC9575677; doi:10.7717/peerj.14118)
Supplement: Supplemental Information 2 [file peerj-10-14118-s002.doc]

Supplemental Table 2. Clustering of gene diversity and virulence diversity when coefficent ＜ 0.21

| Clusters No. | Isolates | Isolates No. |
| --- | --- | --- |
| 1 | L17, L19, L23, L25, L59, L60, H3, H12 | 8 |
| 2 | C2-1, C10-2 | 2 |
| 3 | C13-5, C19 | 2 |
| 4 | C1-3, C11-1, C18, H19 | 4 |
| 5 | C1-2, C2-3, C5-1, C35 | 4 |
| 6 | C6-1, C6-2, C13-3,C13-4, C14, C17 | 6 |
| 7 | L21, L61, L62, L64 | 4 |
| 8 | H16, H45 | 2 |
| 9 | C37, L48 | 2 |
| 10 | C36, C39 | 2 |
| Total | - | 36 |
