# Supplementary figures and images for: Virulence characteristics of Blumeria graminis f. sp. tritici and its genetic diversity by EST-SSR analyses
Source: PeerJ. 2022 Oct 14;10:e14118. doi: 10.7717/peerj.14118 (PMC9575677; doi:10.7717/peerj.14118)

**The original, full-length gel of Fig. 1**


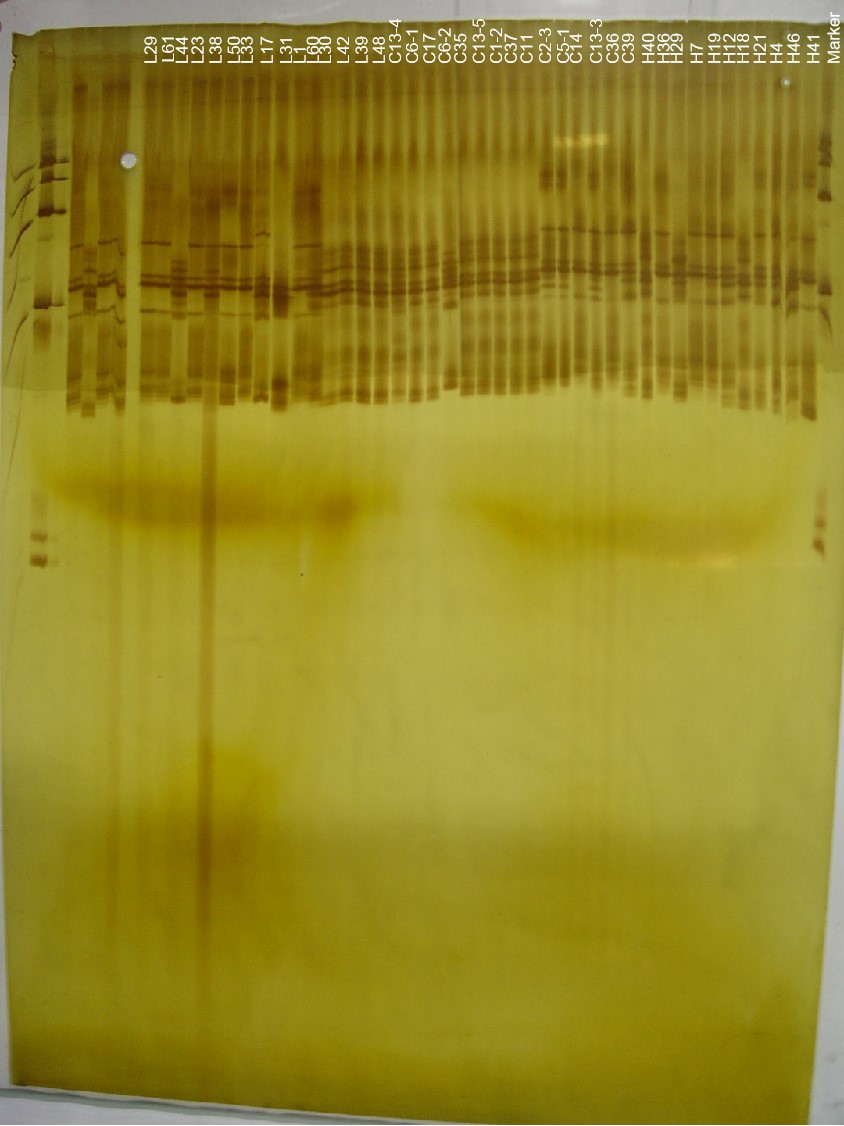


The original, full-length gel of Fig. 2


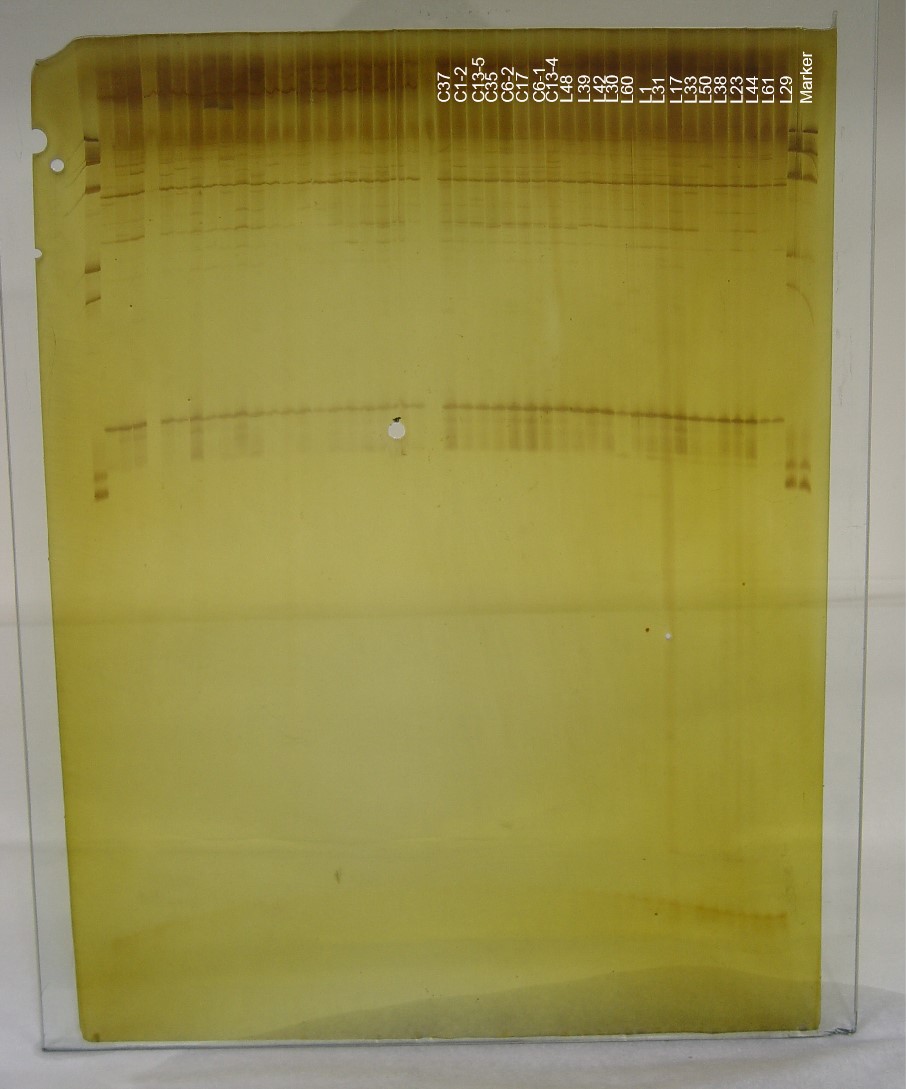

Supplement: Supplemental Information 3 [file peerj-10-14118-s003.doc]
